# Real-time and lightweight detection of grape diseases based on Fusion Transformer YOLO

**DOI:** 10.3389/fpls.2024.1269423

**Published:** 2024-02-23

**Authors:** Yifan Liu, Qiudong Yu, Shuze Geng

**Affiliations:** College of Information Technology Engineering, Tianjin University of Technology and Education, Tianjin, China

**Keywords:** grape diseases detection, YOLO, transformer, lightweight, real-time

## Abstract

**Introduction:**

Grapes are prone to various diseases throughout their growth cycle, and the failure to promptly control these diseases can result in reduced production and even complete crop failure. Therefore, effective disease control is essential for maximizing grape yield. Accurate disease identification plays a crucial role in this process. In this paper, we proposed a real-time and lightweight detection model called Fusion Transformer YOLO for 4 grape diseases detection. The primary source of the dataset comprises RGB images acquired from plantations situated in North China.

**Methods:**

Firstly, we introduce a lightweight high-performance VoVNet, which utilizes ghost convolutions and learnable downsampling layer. This backbone is further improved by integrating effective squeeze and excitation blocks and residual connections to the OSA module. These enhancements contribute to improved detection accuracy while maintaining a lightweight network. Secondly, an improved dual-flow PAN+FPN structure with Real-time Transformer is adopted in the neck component, by incorporating 2D position embedding and a single-scale Transformer Encoder into the last feature map. This modification enables real-time performance and improved accuracy in detecting small targets. Finally, we adopt the Decoupled Head based on the improved Task Aligned Predictor in the head component, which balances accuracy and speed.

**Results:**

Experimental results demonstrate that FTR-YOLO achieves the high performance across various evaluation metrics, with a mean Average Precision (mAP) of 90.67%, a Frames Per Second (FPS) of 44, and a parameter size of 24.5M.

**Conclusion:**

The FTR-YOLO presented in this paper provides a real-time and lightweight solution for the detection of grape diseases. This model effectively assists farmers in detecting grape diseases.

## 1 Introduction

China’s extensive agricultural heritage, spanning over 2000 years, encompasses grape cultivation. Not only it is a significant grape-producing nation but it also stands as the largest exporter of grapes worldwide. Grapes are not only consumed directly but are also processed into various products such as grape juice, raisins, wine, and other valuable commodities, thus holding substantial commercial value (El-Saadony et al., 2022). However, during the grape growth process, susceptibility to diseases can lead to reduced grape yield and significant economic losses (Elnahal et al., 2022). Hence, the timely and effective detection of grape diseases is crucial for ensuring healthy grape growth. Conventionally, the diagnosis of grape diseases predominantly relies on field inspections by agricultural experts (Liu et al., 2022; Ahmad et al., 2023). This approach incurs high costs, has a lengthy cycle, and lacks operational efficiency.

The development of computer vision and machine learning technology provides a new solution for real-time automatic detection of crop diseases (Fuentes et al., 2018, 2019). Traditional machine learning methods in crop diseases identification and positioning have made some valuable experience, such as image segmentation [such as K-means clustering (Trivedi et al., 2022) and threshold method (Singh and Misra, 2017)], feature detection [such as SURF (Hameed and Üstündağ, 2020), KAZE (Rathor, 2021), and MSER blob (Lee et al., 2023)], and pattern recognition [such as KNN (Balakrishna and Rao, 2019), SVM, and bp neural network (Hatuwal et al., 2021; Kaur and Singh, 2021)]. Due to the complexity of image preprocessing and feature extraction, these methods are still ineffective in detection.

Deep learning can automatically learn the hierarchical features of different disease regions without manual design of feature extraction and classifier, with excellent generalization ability and robustness. The detection of crop diseases through CNN has become a new hotspot in intelligent agriculture research. Jiang et al. (2019) proposed a novel network architecture invar-SSD based on VGG-Net and inception to the detection of apple leaf diseases, mAP reached 78.8%. Yang et al. (2023) proposed a SE-VGG16 model uses VGG16 as the basis and adds the SE attention, which classified corn weeds with an average accuracy of 99.67%. Guan et al. (2023) proposed a dise efficient based on the EfficientNetV2 model, achieved an accuracy of 99.80% on the plant disease and pest dataset. The above three methods are merely applicable for simple classification tasks. However, when it comes to detection tasks, the prevailing approach currently in use is YOLO. Liu and Wang (2020) proposed an improved YOLOv3 algorithm to detect tomato diseases and insect pests. Results show that the detection accuracy is 92.39%, and the detection time is 20.39 ms. Wang et al. (2022) proposed a lightweight model based on the improved YOLOv4 to detect dense plums in orchards. Compared with YOLOv4 model, the model size is compressed by 77.85%, the parameters are only 17.92%, and the speed is accelerated by 112%. Kuznetsova et al. (2020) designed harvesting robots based on a YOLOv3 algorithm, apple detection time averaged 19 ms with 90.8% recall, and 7.8% False Positive Rate (FPR). Qi et al. (2021) proposed a highly fused, lightweight detection model named the Fusion-YOLO model to detect the early flowering stage of tea chrysanthemum. Huang et al. (2021) used the YOLOv5 algorithm to detect the citrus collected by UAV, the detection accuracy rate was 93.32%. Qiu et al. (2022) used YOLOv5 for detecting citrus greening disease. The F1 scores for recognizing five symptoms achieved 85.19%. Zhou et al. (2022) proposed an improved YOLOX-s algorithm. Compared with the original YOLOX-s, the model improved the detection Average precision (AP) of kiwifruit by 6.52%, reduced the number of parameters by 44.8% and upgraded the model detection speed by 63.9%. Soeb et al. (2023) used YOLOv7 for five tea leaf diseases in natural scene, which validated by detection accuracy 97.3%, precision 96.7%, recall 96.4%, mAP 98.2%, and F1-score 0.965, respectively.

The application of machine learning and deep learning in crop disease detection in recent years is summarized. Deep learning, especially CNN, has also made some contributions to grape disease detection. Ji et al. (2020) designed the United Model and selected 1,619 images of healthy and three kinds of diseased grape leaves in Plant village, with detection accuracy up to 98.57%. However, it should be noted that all the data were obtained from laboratory samples, and no comparative experiments were conducted in a natural environment. Sanath Rao et al. (2021) used a pre-trained AlexNet to classify grapes and mango leaf diseases, achieved accuracy of 99% and 89% for grape leaves and mango leaves, respectively. Ji et al. (2020) proposed a united CNN architecture based on InceptionV3 and ResNet50 and can be used to classify grape images into four classes, achieved average validation accuracy of 99.17% and test accuracy of 98.57%. Adeel et al. (2022) proposed a entropy-controlled CNN to identify grape leaf diseases at the early stages, achieved an accuracy of 99%. Lu et al. (2022) proposed a Ghost-conv. and Transformer networks for diagnosing 11 classes grape leaf and pest, reached 180 frames per second (FPS), 1.16 M weights and 98.14% accuracy. After adding Transformer and Ghost-conv., the performance is improved significantly, but only the identification work is done. Xie et al. (2020) presented a Faster DR-IACNN model with higher feature extraction capability, achieved a precision of 81.1% mAP, and the detection speed reaches 15.01 FPS. The above two methods only detect grape leaf diseases. Sozzi et al. (2022) evaluated six versions of the YOLO (YOLOv3, YOLOv3-tiny, YOLOv4, YOLOv4-tiny, YOLOv5x, and YOLOv5s) for real-time bunch detection and counting in grapes. Pinheiro et al. (2023) presented three pre-trained YOLO models (YOLOv5x6, YOLOv7-E6E, and YOLOR-CSP-X) to detect and classify grape bunches as healthy or damaged by the number of berries with biophysical lesions, highlighting YOLOv7 with 77% of mAP and 94% of the F1-score. Both of the aforementioned methods solely utilized YOLO for grape bunch detection and did not involve disease detection. Zhu et al. (2021) proposed YOLOv3-SPP network for detection of black rot on grape leaves, applied in field environment with 86.69% precision and 82.27% recall. Zhang Z. et al. (2022) proposed a YOLOv5-CA, which highlights the downy mildew disease–related visual features to achieve an mAP of 89.55%. Both methods employed YOLO for the detection of a single disease in grapes. We have listed the advantages and disadvantages of different methods for plant disease detection in **Table 1**.

**Table 1:** Comparison of the advantages and disadvantages of different methods.

Method	Advantage	Disadvantage
Machine learning	- Less data and computing resources. - High interpretability.	- Difficult to handle complex problems. - Poor detection accuracy.
Deep learning (classification)	- The model can automatically learn image feature representations. - High detection accuracy.	- More data and computing resources. - The task is relatively simple; the practical value is limited.
Deep learning (detection)	- Higher accuracy and generalization hold significant practical value. - End-to-end, one-stage models (YOLO) are easy to implement.	- Additional data, annotations, and computing resources are necessary. - Balancing detection accuracy and speed is a challenging task.

There are also several challenges in grape disease detection: (1) grape fruits and inflorescence are small and dense, making it difficult to detect the incidence area, which can be very small. (2) Photos taken in natural scenes are susceptible to external interference. (3) The model needs to balance detection accuracy with lightweight requirements for deployment and real-time performance. To address these challenges, this paper proposes a real-time detection model based on Fusion Transformer YOLO (FTR-YOLO) for grape diseases. The main contributions of this paper are summarized as follows:

- Regarding the issue of limited detection of disease types in other models and the detection under non-natural environments, we have collected four grape diseases (anthracnose, grapevine white rot, gray mold, and powdery mildew) datasets in natural environments, covered different parts such as leaves, fruits, and flower inflorescence. The primary source of the dataset comprises RGB images acquired from plantations situated in North China.
- In backbone, we integrate learnable downsampling layer (LDS), effective squeeze and excitation (eSE) blocks, and residual connections based on VoVnet, effectively improving the ability of network to extract feature information. In neck component, an improved real-time Transformer with two-dimensional (2D) position embedding and single-scale Transformer encoder (SSTE) are incorporated to the last feature map to accurate detection of small targets. In head component, the Decoupled Head based on the improved Task-Aligned Predictor (ITAP) is adopted to optimize detection accuracy.
- To address the challenges with deploying application using models that have a large capacity and slow inference speed, we replace the convolution with ghost module in the model, abandon Transformer decoder, and adopt more efficient SSTE with VoVnet-39 of fewer layers to ensure the lightweight and detection speed.

The rest of the article is organized as follows: **Section 2** explicates the datasets and experimental settings and the network architecture and improvement of FTR-YOLO. **Section 3** presents the evaluation of the experimental performance and analyses. Discussions of the performance are presented in **Section 4**. Last, **Section 5** offers conclusions and suggestions for future work.

## 2 Materials and methods

### 2.1 Experimental dataset building

In the process of building grape diseases detection dataset, smartphone is used to collect photos in the local orchard. The photos are taken in different time periods, weather conditions, and scenes. The labeling tool is used to mark the images, the region of interest by manually marking the rectangle, and then generated the configuration file automatically.

Data augmentation is employed to expand the number of images within the training dataset. The methods include random flipping, Gaussian blur, affine transformation, image interception, filling, and so forth. The network model is designed to enhance randomly selected images by one or several operations.

The number of samples for each category is shown in **Table 2**. Through data enhancement, the dataset is expanded to 4,800 images. The ratio of training set and test set is 8:2.

**Table 2:** The number of samples for each disease type.

Disease	Sample size	Number of labeled samples (bounding box)	Percent of bounding box samples
Anthracnose	1200	4587	20.53%
White rot	1200	6025	26.97%
Gray moid	1200	5160	23.09%
Powdery mildew	1200	6571	29.41%
Total	4800	22343	100%

**Figure 1:**
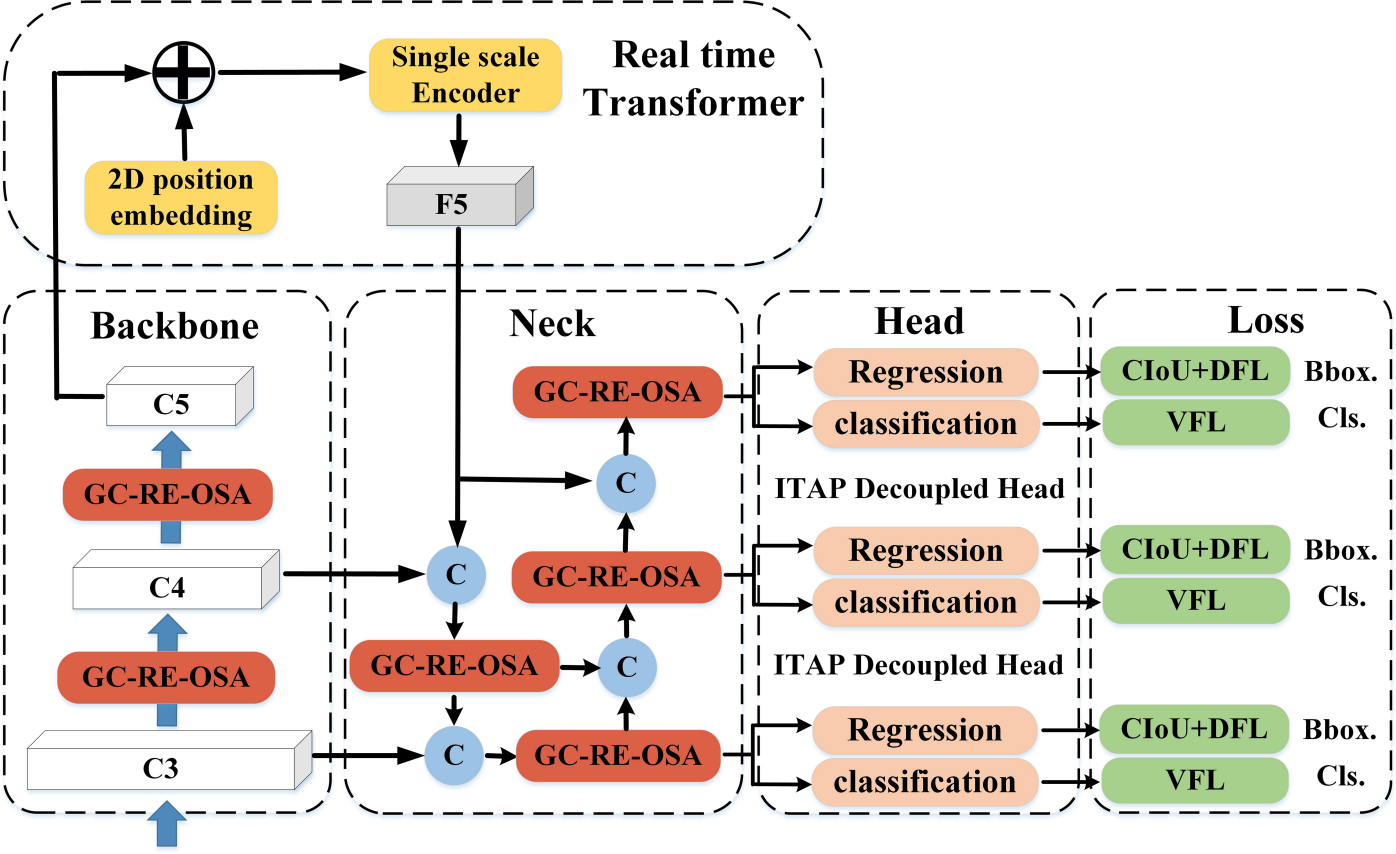
The architecture of FTR-YOLO.

The overall structure of FTR-YOLO is shown in **Figure 1**. The primary innovations of the model are represented by streamlined modules. For comprehensive details, please consult the detailed illustrations provided in **Sections 2.2–2.4**.

### 2.2 Backbone of FTR-YOLO

In backbone component, a lightweight high-performance VoVnet (LH-VoVNet) (Zhao et al., 2022) network is used. The proposed net adds the LDS Layer, eSE attention module (Long et al., 2020) and residual connection on the basis of One-Shot Aggregation (OSA) module. Also, the Conv. layer is replaced with Ghost Module (Zhang B. et al., 2022) to further lightweight the network. The LH-VoVNet has shorter computation time and higher detection accuracy compared with other common backbone, which is more suitable for grape disease detection tasks.

#### 2.2.1 VoVNet

**Figure 2:**
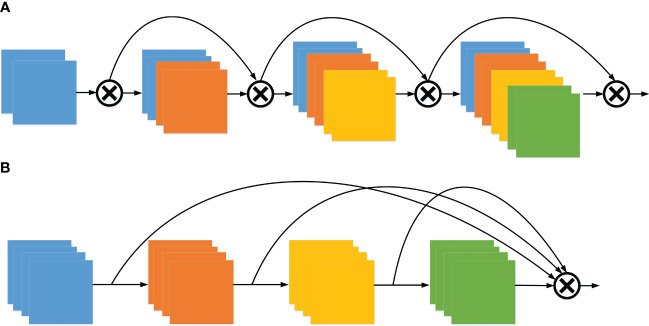
The architecture of DenseNet and VoVNet. **(A)** Dense aggregation (DenseNet) and **(B)** One-shot aggregation (VoVNet).

One of the challenges with DenseNet (Jianming et al., 2019) is that the dense connections can become overly cumbersome. Each layer aggregates the features from the preceding layers, leading to feature redundancy. Furthermore, based on the L1 norm of the model weights, it is evident that the middle layer has minimal impact on the final classification layer, as shown in **Figure 2A**. Instead, this information redundancy is a direction that can be optimized, so the OSA module is adopted, as shown in **Figure 2B**. Simply put, the OSA aggregates all the layers up to the final one, effectively addressing the prior issue encountered with DenseNet. Since the number of input channels per layer is fixed, the number of output channels can be consistent with the input to achieve the minimum MAC, and the 1 × 1 Conv. layer is no longer required to compress features, the OSA module is computationally efficient.

#### 2.2.2 LDS layer

**Figure 3:**
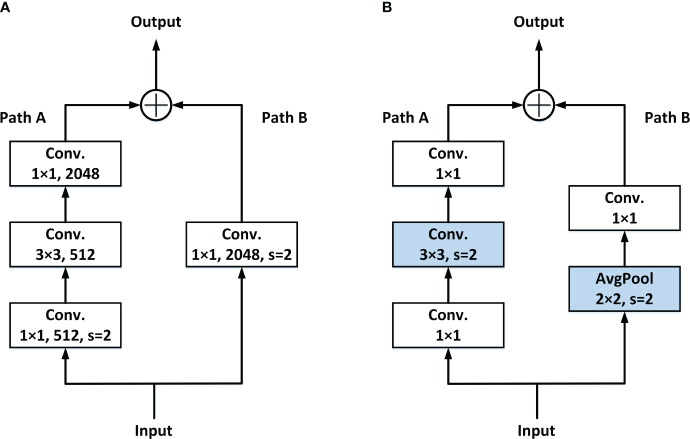
Two different methods of downsampling. **(A)** Conv. downsampling and **(B)** LDS downsampling.

At present, in common networks, the steps of downsampling feature maps are usually completed at the first Conv. of each stage. **Figure 3A** shows the general Residual block. In Path A, once the input data are received, it undergoes a 1 × 1 Conv. with a stride of 2. This operation leads to a loss of 3/4 of the information in the input feature maps.

To solve this problem, the LDS layer is adopted. The downsampling is moved to the following 3 × 3 Conv. in Path A, and the identity part (Path B) downsampling is done by the added avg-pool, so as to avoid the loss of information caused by the simultaneous appearance of 1 × 1 Conv. and stride. Details are shown in **Figure 3B**.

#### 2.2.3 RE-OSA module

The pivotal element of the VoVnet lies in the OSA module as described in **Section 2.2.1**. While the performance of the OSA module is not enhanced, it offers lower MAC and improved computational efficiency. Therefore, this paper adds eSE block and residual connection in OSA module to further enhance features and improve detection accuracy, called RE-OSA module.

The core idea of SE Block is to learn the feature weight according to loss through the network (Hu et al., 2018), so that the effective feature map has a larger weight and the rest of the feature map has a smaller weight to train the model to achieve better results. The SE module squeezes the entire spatial features on a channel into a global feature by global average pooling, then two fully connected (FC) layers are used to concat the feature map information of each channel. Assume that the input feature map $X_{i}\in R^{C\times W\times H}$ , the channel attention map $A_{ch}(X_{i})\in R^{C\times 1\times 1}$ is computed in **Equations 1**, **2**.


(1) $$A_{ch}(X_{i})=\mathrm{\sigma (}W_{c}(\mathrm{\delta (}W_{c/r}(F_{gap}(X_{i})))))$$

(2) $$F_{gap}(X)=\frac{1}{WH}\sum_{i,j=1}^{W,H}X_{i,j}$$

Where $F_{gap}$ is channel-wise global average pooling, $W_{c/r}$ , $W_{c}\in R^{C\times 1\times 1}$ are weights of two FC layers, σ denotes ReLU activation function, δ denotes sigmoid activation function.

In SE block, to avoid the computational burden of such a large model, reduction ratio *r* is used in the first FC layer to reduce the input feature channels from *c* to *c/r*. The second FC layer needs to expand the reduced number of channels to the original channel *c*. In this process, the reduction of channel dimensions leads to the loss of channel information.

Therefore, we adopt eSE that uses only one FC layer with *c* channels instead of two FC layers without channel dimension reduction, which rather maintains channel information and in turn improves performance. In this paper, the ReLU/sigmoid activation function in the module is replaced by the SiLU function with better performance in YOLOv7 (Wang et al., 2023). The eSE is computed in **Equations 3**, **4**:


(3) $$A_{eSE}=\vartheta (W_{c}(F_{gap}(X_{i})))$$

(4) $$X_{refine}=A_{eSE}⊗X_{i}$$

where ϑ denotes SiLU activation function. As a channel attentive feature descriptor, the $A_{eSE}\in R^{C\times 1\times 1}$ is applied to the diversified feature map *X_i_* to make the diversified feature more informative. Finally, the refined feature map *X_refine_* is obtained by channel-wise multiplication *A_eSE_* and *X_i_*.

#### 2.2.4 Lightweight with ghost convolution

It can be seen from **Section 2.2.3** that Conv. layer appears most frequently in VoVNet. As a result, the whole network has a large amount of computation and parameter volume, which is not conducive to lightweight deployment.

To solve this problem, this paper adopts a structure—Ghost Module, which can generate a large number of feature graphs with cheap operations. This method can reduce the amount of computation and parameter volume on the basis of ensuring the performance ability of the algorithm.

In the feature map extracted by the mainstream deep neural networks, the rich and even redundant information usually ensures a comprehensive understanding of the input data. These redundancies are called ghost maps.

The ghost module consists of two parts. One part is the feature map generated by the ordinary Conv. The other part is the ghost maps generated by simple linear operation Φ. It is assumed that the input feature map of size *h*×*w*×*c* is convolved with n sets of kernels of size *k*×*k*, and the output feature map of size *h'*×*w'*×*n*. In the ghost model, m groups of *k*×*k* kernels are convolved with input to generate the identity maps of size *h'*×*w'*×*m*, after which the identity maps are linearly transformed by depth-wise convolution (*k*=5) to produce ghost maps. Finally, identity maps are concated with ghost maps to generate ghost convolution. The ghost convolution acceleration ratio *r_s_* and compression ratio *r_c_* are calculated compared with ordinary convolution, as shown in Equations 5, 6.


(5) $$r_{s}=\frac{n\cdot h\prime \cdot w\prime \cdot c\cdot k\cdot k}{\frac{n}{s}\cdot h\prime \cdot w\prime \cdot c\cdot k\cdot k+(s-1)\cdot \frac{n}{s}\cdot h\prime \cdot w\prime \cdot c\cdot d\cdot d}\approx s$$

(6) $$r_{c}=\frac{n\cdot c\cdot k\cdot k}{\frac{n}{s}\cdot c\cdot k\cdot k+(s-1)\cdot \frac{n}{s}\cdot c\cdot d\cdot d}\approx s$$

where the numerator is the complexity of ordinary convolution. The denominator is the complexity of ghost module. *s* is the total mapping generated by each channel (one identity map and s-1 ghost maps), *c* is the number of input feature maps, generally s≪c ; *n*/*s* refers to the identity map output by general convolution; *d*×*d* is the average kernel size of depth-wise Conv. and has a similar size to *k*×*k*.

Equations 5, 6 show that, compared with ordinary Conv., Ghost-conv. greatly reduces the amount of computation and the number of parameters.

**Figure 4:**
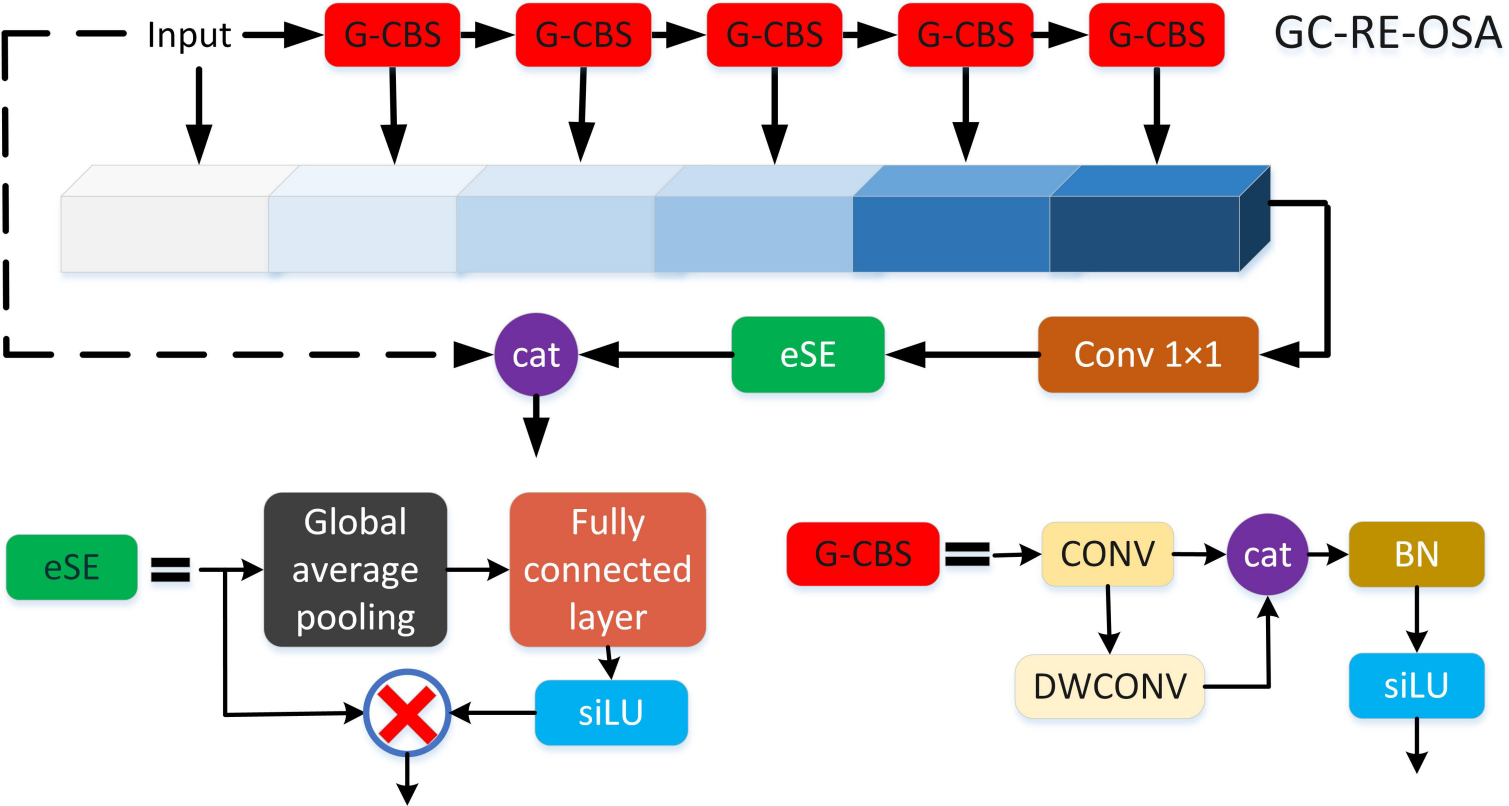
The structure of GC-RE-OSA module.

Finally, GC-RE-OSA module replaced 3 × 3 Conv. in RE-OSA module (**Section 2.2.3**) with Ghost-conv. The structure of GC-RE-OSA is shown in **Figure 4**.

The specific structure of LH-VoVnet can be found in **Table 3**. LH-VoVNet comprises a stem block that consists of three 3 × 3 Conv. layers, followed by GC-RE-OSA modules implemented in four stages. At the start of each stage, an LDS with a stride of 2 is utilized (**Section 2.2.2)**. The model achieves a final output stride of 32. For more details, please refer to **Sections 2.2.3** and **2.2.4**.

**Table 3:** The specific structure of LH-VoVnet.

Type	Output stride	Stage	Output channel
Stem	2 2 2	3×3 Ghost-conv., 64, Stride = 2 3×3 Ghost-conv., 64, Stride = 1 3×3 Ghost-conv., 128, Stride = 1	64
Stage 1	4	LDS Layer ×1, GC-RE-OSA×1	128
Stage 2	8	LDS Layer ×1, GC-RE-OSA×1	256
Stage 3	12	LDS Layer ×1, GC-RE-OSA×2	512
Stage 4	32	LDS Layer ×1, GC-RE-OSA×2	1024

### 2.3 Neck of FTR-YOLO

Indeed, the Transformer model relies on a global attention mechanism that requires substantial computational resources for optimal performance (Carion et al., 2020). Consequently, it becomes crucial to address this issue effectively. To mitigate this concern, we eschew the initial image or multi-layer feature maps as input and instead incorporate only the final feature map obtained from the backbone. This is then directly connected to the neck. Additionally, we select only two improved modules of Position Embedding and Encoder.

Within the neck component, we utilize the current optimal dual-flow PAN + FPN structure and enhance it through integration with the GC-RE-OSA module introduced in this paper.

#### 2.3.1 Real-time transformer

To enhance the detection accuracy, an enhanced global attention mechanism based on the Vision Transformer (ViT) is introduced. This modification takes into consideration that some grape diseases may share similarities, while others have limited occurrence areas. By incorporating this improved global attention mechanism, the detection accuracy can be further improved in detecting different grape diseases.

The current common detection transformer (DETR) algorithms extract the last three layers of feature maps (C3, C4, and C5) from the backbone network as the input. However, this approach usually has two problems:

1. Previous DETRs, such as deformable DETR (Zhu et al., 2020), flatten multi-scale features, and concatenate them into a single long-sequence vector. This approach not only enables effective interaction between the different scale features but it also introduces significant computational complexity and increases the time required for processing.
2. Compared to the shallower C3 and C4 features, the deepest layer C5 feature has deeper, higher level, and richer semantic features. These semantic features are more useful for distinguishing different objects and are more desirable for Transformer. Shallow features do not play much of a role due to the lack of better semantic features.

To address these issues, we only select the C5 feature map output by the backbone network as the input for the Transformer. To retain key feature information as much as possible, we replaced the simple flattening of feature maps into a vector with a 2D encoding in the Position Embedding module (Wu et al., 2021). Additionally, a lightweight single-scale Transformer encoder is adopted.

The Multi-Head Self-Attention (MHSA) aggregation in Transformer combines input elements without differentiating their positions; thus, Transformer possess permutation invariance. To alleviate this issue, we need to embed spatial information into the feature map, which requires adding 2D position encoding to the final layer feature map. Specifically, the original sine and cosine positional encodings in Position Embedding are respectively extended to column and row positional encodings, and concatenated with them finally.

After the feature map is processed by 2D position embedding, we use a single-scale Transformer Encoder, which only contains one Encoder layer (MHSA + Feed Forward network) to process the output of Q, K, and V at three scales. Note that the three scales share one SSTE and, through this shared operation, the information of the three scales can interact to some extent. Finally, the processing results are concatenated together to form a vector, which is then adjusted back to a 2D feature map, denoted as F5. In the neck part, C3, C4, and F5 are sent to dual-flow PAN + FPN for multi-scale feature fusion. See **Figure 1** for details.

#### 2.3.2 Dual-flow PAN + FPN

**Figure 5:**
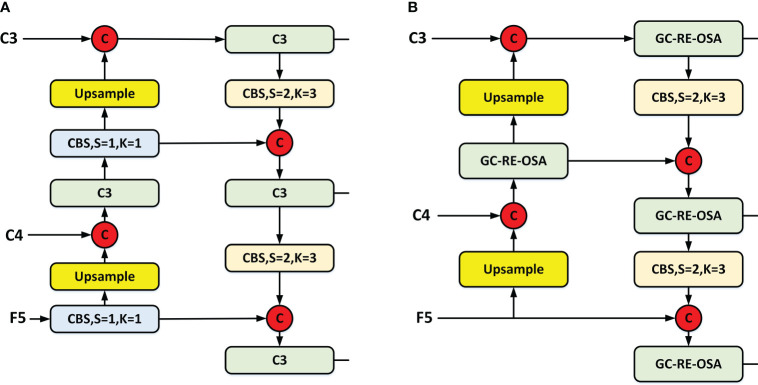
Two different neck structures. **(A)** YOLOv5 neck and **(B)** ours.

In order to achieve better information fusion of the three-layer feature maps (C3, C4, and F5), our enhanced neck implements a dual-stream PAN + FPN architecture, which is featured in the latest YOLO series. In addition to this, we have introduced GC-RE-OSA module to ensure faster detection speed while preserving accuracy. A comparison between YOLOv5 (Jocher et al., 2021) (**Figure 5A**) and our enhanced neck structure (**Figure 5B**) is provided. Our improved architecture substitutes the C3 module with the GC-RE-OSA module and eliminates the Conv. prior to upsampling. This enables direct utilization of features output from diverse stages of the backbone.

### 2.4 Head of FTR-YOLO

For the Head component, we have employed Decoupled Head to perform separate classification and regression tasks via two distinct convolutional channels. Furthermore, our architecture includes the ITAP within each branch, which enhances the interaction between the two tasks.

Object detection commonly faces a task conflict between classification and localization. While decoupled head is successfully applied to SOTA YOLO model in YOLOX (Ge et al., 2021), v6 (Li et al., 2023), v7 (Wang et al., 2023), and v8 (Terven and Cordova-Esparza, 2023), drawing lessons from most of the one-stage and two-stage detectors, single-stage detectors perform classification and localization tasks in parallel using two independently functioning branches. However, this dual-branch approach may lack interaction, resulting in inconsistent predictions during execution.

**Figure 6:**
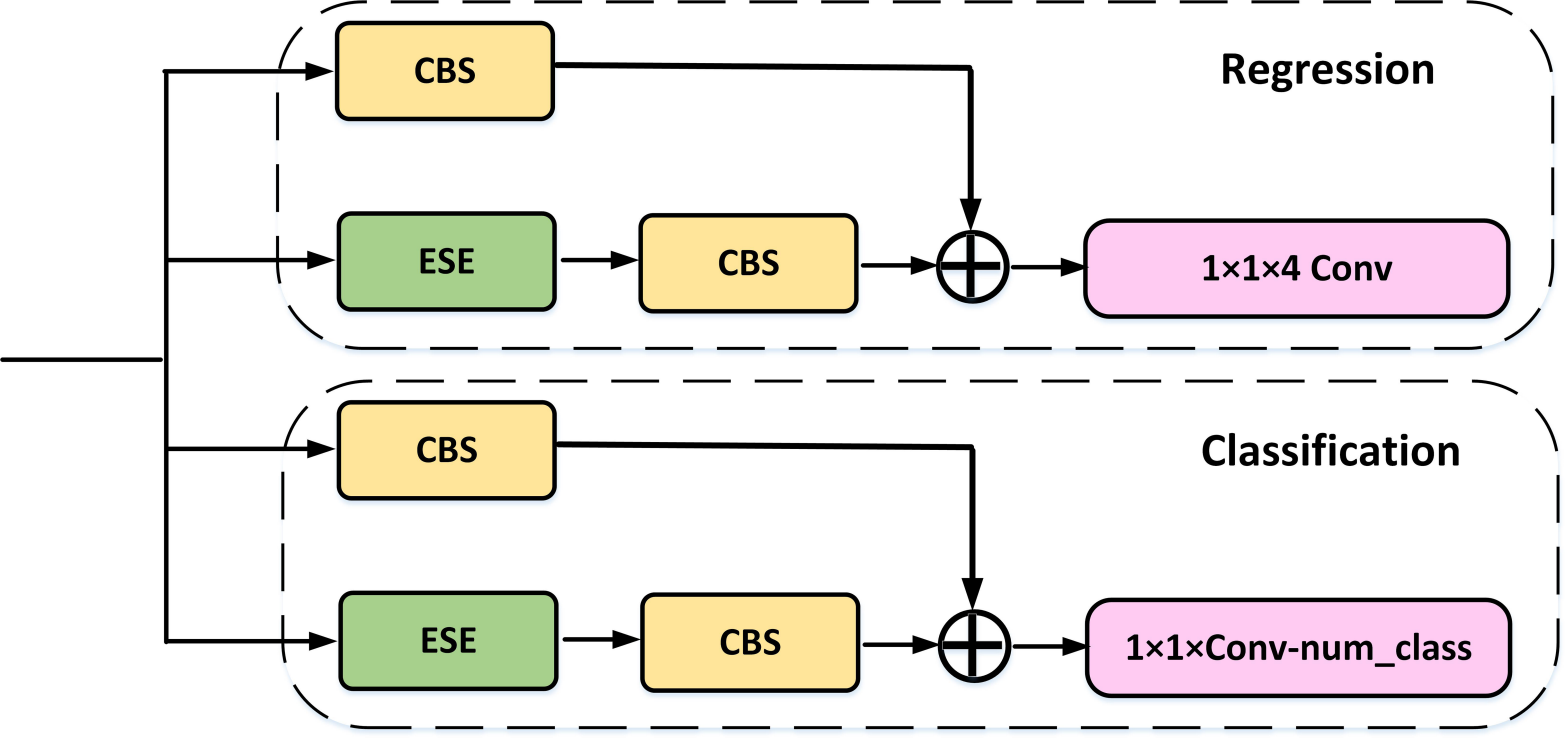
ITAP decoupled head structures.

To address this issue, we drew inspiration from the TAP in TOOD (Feng et al., 2021) and made some improvements to maintain accuracy while improving speed. As shown in **Figure 6**, the ITAP uses eSE to replace the layer attention in TOOD. To further enhance efficiency, we incorporated a more efficient Convolution+BN layer+Silu (CBS) module before the shortcut. Moreover, during the training phase, we utilized different loss for the two branches.

### 2.5 Label assignment and loss

The loss calculation in our study employed the label assignment strategy. SimOTA is employed in YOLOX, v6 and v7 to enhance their performance. Task alignment learning (TAL) proposed in TOOD is used in YOLOv8. This strategy entails selecting positive samples based on the weighted scores of the classification and regression branches within the loss function. For the classification branch, we utilize the varifocal loss (VFL) (Zhang et al., 2021), while for the regression branch, the distribution focal loss (DFL) (Li et al., 2020) is employed. Furthermore, we incorporate the Complete-IoU (CIoU) Loss. The combination of these three losses is achieved through weighted proportions.

VFL utilizes the target score to assign weight to the loss of positive samples. This implementation significantly amplifies the impact of positive samples with high IoU on the loss function. Consequently, the model prioritizes high-quality samples during the training phase while de-emphasizing the low-quality ones. Similarly, both approaches utilize IoU-aware classification score (IACS) as the target for prediction. This enables effective learning of a combined representation that includes both classification score and localization quality estimation. By employing DFL to tackle the uncertainty associated with bounding boxes, the network gains the ability to swiftly concentrate on the distribution of neighboring regions surrounding the target location. See Equation 7 for details.


(7) $$Loss=\frac{\alpha \cdot loss_{VFL}+\beta \cdot loss_{CIoU}+\gamma loss_{DFL}}{\sum_{i}^{N_{pos}}\hat{t}}$$

where $\hat{t}$ denotes the normalized score used in TOOD, α, β, and γ represent different weights.

## 3 Experimental results

The experimental hardware environment is configured with INTEL I7-13700 CPU, 32GB RAM, and GEFORCE RTX3090 graphics. The operating system is Windows10 professional edition, the programming language is Python 3.8, and the acceleration environment is CUDA 11.1 and CUDNN 8.2.0. The training parameters of the training process used in the experiment are shown in **Table 4**.

**Table 4:** The implementation details of training parameters.

Parameter	Value	Parameter	Value
Optimizer	AdamW	Weight decay	0.0005
Learning rate	0.001	Momentum	0.937
Batch size	8	warmup steps	300
Image size	640*640	Epochs	200
NMS threshold	0.7	EMA decay	0.9998

### 3.1 Ablation study on backbone

The improved network is composed of backbone, neck, and head, so the influence of the improvement of each part on the model performance should be verified respectively.

In this paper, the LH-VoVNet is verified through experiment. The improvements include (1) the LDS layer is used for downsampling. (2) By adding eSE block and RE-OSA module. (3) The Conv. is replaced with Ghost Module to further lightweight the network. The results of the ablation study are shown in **Table 5**.

**Table 5:** The results of the ablation study of backbone components.

Methods	mAP@0.5	Params(M)	FPS
VoVnet	84.62	49.0	38
+LDS layer	85.68	49.4	37
+RE-OSA module	86.04	53.5	24
+Ghost-conv.	84.93	**18.3**	**68**
LH-VoVNet	**86.79**	24.7	56

Bold values represents the optimal values.

On the basis of VoVnet, compared by adding LDS layer/RE-OSA module improves accuracy by 1.06%/1.42% mAP. By replacing Ghost-conv., the number of parameters in the network is greatly reduced (−62.7%), the FPS is significantly improved (+78.9%), and the detection performance is also slightly improved (+0.31%). Finally, the integration of these three components shows that mAP 86.79% (+2.17%) is optimal, Params 24.7MB (−50.1%) and FPS 56 (+47.4%), achieve lightweight and real-time in backbone.

### 3.2 Ablation study on neck

To verify the effectiveness of the proposed neck, we evaluate the indicators of the set of variants designed in **Section 2.3**, including mAP, number of parameters, latency and FPS. The backbone used in the ablation experiment is LH-VoVNet. The improvements include the following: (1) Only the C5 feature map output by the backbone as the input is selected for the Transformer. (2) The real-time Transformer only includes 2D position embedding and SSTE to further lightweight the network. (3) The C3 module is replaced with GC-RE-OSA module. The parameters for the Transformer Encoder are as follows: num of head = 8, num of encoder layers = 1, hidden dim = 256, dropout = 0.1, activation = relu.

The experimental results are shown in **Table 6**. On the basis of YOLOv5 neck, by adding real-time Transformer delivers 1.41% AP improvement, while increasing the number of parameters by 4.5%, the latency by 47.2%, decreasing the FPS by 17.9%. This demonstrates the effective enhancement of detection accuracy by Transformer while maintaining a high degree of lightweight and real-time performance. By adding GC-RE-OSA module delivers 0.45% AP improvement, the number of parameters experienced a slight increase of 4.5%, the latency decreases by 25.0%, and the FPS increase by 8.9%. This shows that the module not only enables lightweight networking but also enhances performance. Finally, the integration of these two components shows that mAP 88.85% (+2.06%) is optimal, Params 22.5MB (−8.9%), Latency 56.3ms (+7.2%), and FPS 49 (−12.5%). The improved neck further enhances network detection performance and lightweight, albeit with a slight fluctuation in FPS and Latency that has negligible impact on real-time detection.

**Table 6:** The results of the ablation study of neck components.

Methods	mAP@0.5	Params(M)	Latency(ms)	FPS
YOLOv5 neck	86.79	24.7	52.5	56
+Real time Transformer	88.20	25.8	77.3	46
+GC-RE-OSA module	87.22	**20.3**	**39.4**	**61**
Ours neck	**88.85**	22.5	56.3	49

Bold values represents the optimal values.

### 3.3 Ablation study on head and loss

To verify the effectiveness of the proposed head, we evaluate the indicators of the set of variants designed in **Sections 2.4 and 2.5**, including mAP, number of parameters, latency, and FPS. We conduct this experiment on above-modified model, which uses LH-VoVNet, improved neck, and YOLOv5 head as the baseline. The parameters for the TAL are as follows: topk = 13, alpha = 1.0, and beta = 6.0. Similarly, for the SimOTA Assigner, the parameters are center_radius = 2.5 and topk = 10. In Equation 7, the weights assigned to the three losses are as follows: VFL (α = 1.0), CIoU (β = 2.5), and DFL (γ = 0.5). The experimental results are shown in **Table 7**.

**Table 7:** The results of the ablation study of head & loss components.

Methods	mAP@0.5	Params(M)	Latency(ms)	FPS
YOLOv5 head	88.85	**22.5**	**56.3**	**49**
+ITAP decoupled head	89.46	23.9	60.0	45
+SimOTA	89.12	23.0	57.1	48
+TAL	89.91	23.1	57.3	48
Ours head	**90.67**	24.5	61.5	44

Bold values represents the optimal values.

On the basis of YOLOv5 head, by adding ITAP Decoupled Head delivers 0.61% AP improvement, while increasing the number of parameters by 6.2%, the latency by 6.6%, decreasing the FPS by 8.2%. This indicates that the improved head has minimal impact on parameter and computational speed, while simultaneously enhancing detection accuracy. By adding SimOTA delivers 0.27% AP improvement, the number of parameters/Latency/FPS experience a slight fluctuation by +2.2%/+1.4%/−2.0%. By adding TAL delivers 1.06% AP improvement, the number of parameters/Latency/FPS experience a slight fluctuation by +2.7%/+1.8%/−2.0%. After comparing the label assignments of SimOTA and TAL, it was found that TAL exhibited superior performance, thus making it the preferred choice for our paper. Finally, we adopted a hybrid methodology comprising ITAP Decoupled Head+TAL, resulting in an optimized mAP of 90.67% (+1.82%). Additionally, there was an augmentation in the model’s parameters and Latency to 24.5MB (+8.9%) and 61.5 (+9.2%), respectively, while the FPS decreased to 44 (−10.2%).

### 3.4 Comparison with other detectors

**Table 8** compares FTR-YOLO with other real-time detectors (YOLOv5, YOLOv6, YOLOv7, YOLOv8, and PP-YOLOE) and Vision Transformer detector (DINO-DETR).

**Table 8:** The comparison results of different methods.

Method	Size	Params(M)	AP for each category*				mAP@0.5	FPS	*p*-value
			1	2	3	4			
Yolo V5	640*640	46.3	87.42	76.03	85.29	88.18	84.23	40	< 0.01
Yolo V6	640*640	59.0	90.70	88.59	80.37	90.14	87.54	37	< 0.01
Yolo V7	640*640	36.6	89.51	90.44	84.23	91.26	88.86	41	< 0.01
Yolo V8	640*640	43.3	89.83	88.47	85.30	92.08	88.92	**44**	< 0.01
PP-YOLOE	640*640	52.2	88.15	78.59	84.42	91.84	85.75	41	< 0.01
DINO-DETR	800*1333	47.4	**91.79**	90.58	**88.76**	**93.35**	**91.12**	2	——
FTR-YOLO	640*640	**24.5**	90.73	**90.67**	88.54	92.74	90.67	**44**	< 0.01

*In **Table 8**, 1, 2, 3, and 4 represent the four types of detected diseases: 1, anthracnose; 2, grapevine white rot; 3, gray mold; 4, powdery mildew. Bold values represents the optimal values.

Compared to real-time detectors YOLOv5/YOLOv6/YOLOv7/YOLOv8/PP-YOLOE, FTR-YOLO significantly improves accuracy by 6.44%/3.13%/1.81%/1.75%/4.92% mAP, increases FPS by 10.0%/18.9%/7.3%/0.0%/7.3%, and reduces the number of parameters by 47.1%/58.5%/33.1%/43.4%/53.1%. Even among the AP metrics for the four categories, the FTR-YOLO algorithm consistently demonstrates the best performance. Additionally, the differences in AP values among the four disease categories are relatively small, indicating that the FTR-YOLO algorithm exhibits good robustness. This demonstrates the superior performance of FTR-YOLO compared to the state-of-the-art YOLO detectors in terms of accuracy, speed, and lightweight.

In order to determine the statistical significance of the differences between various algorithms, we performed four independent repeated experiments for each algorithm. A *t* test was employed, and the *p*-values for mAP among different algorithms were computed. Due to substantial variations in parameters, including image input size and training epochs, between the DINO-DETR algorithm and other detection algorithms, it was excluded from the statistical analysis. The experimental results reveal that the *p*-values comparing different algorithms are considerably small, all well below 0.01, signifying noteworthy variances between the algorithms.

Compared to DINO-DETR, the number of parameters/mAP/FPS experience a fluctuation by −48.3%/−0.45%/+2100.0%. This observation highlights that, while DINO achieves a slightly higher mAP of 0.45% compared to FTR-YOLO, it fails to meet real-time requirements due to its significantly lower FPS (2). Furthermore, there is no discernible advantage in terms of model lightweight.

### 3.5 Object size sensitivity analysis

Different disease types, periods, and locations result in different characteristics and sizes. The improved network proposed in this paper effectively enhances the detection accuracy in small object scenario. In order to verify the detection effect of the small object detection performance, the test dataset is divided into five groups based on the size of the disease area. The, 0%–10%, 10%–20%, 20%–40%, 40%–60%, and 60%–90%, five groups are named with different labels: XS, S, M, L, and XL, which represent the size of different objects. The comparison of the detection accuracy of six common algorithms with FTR-YOLO for five different sizes.

**Figure 7:**
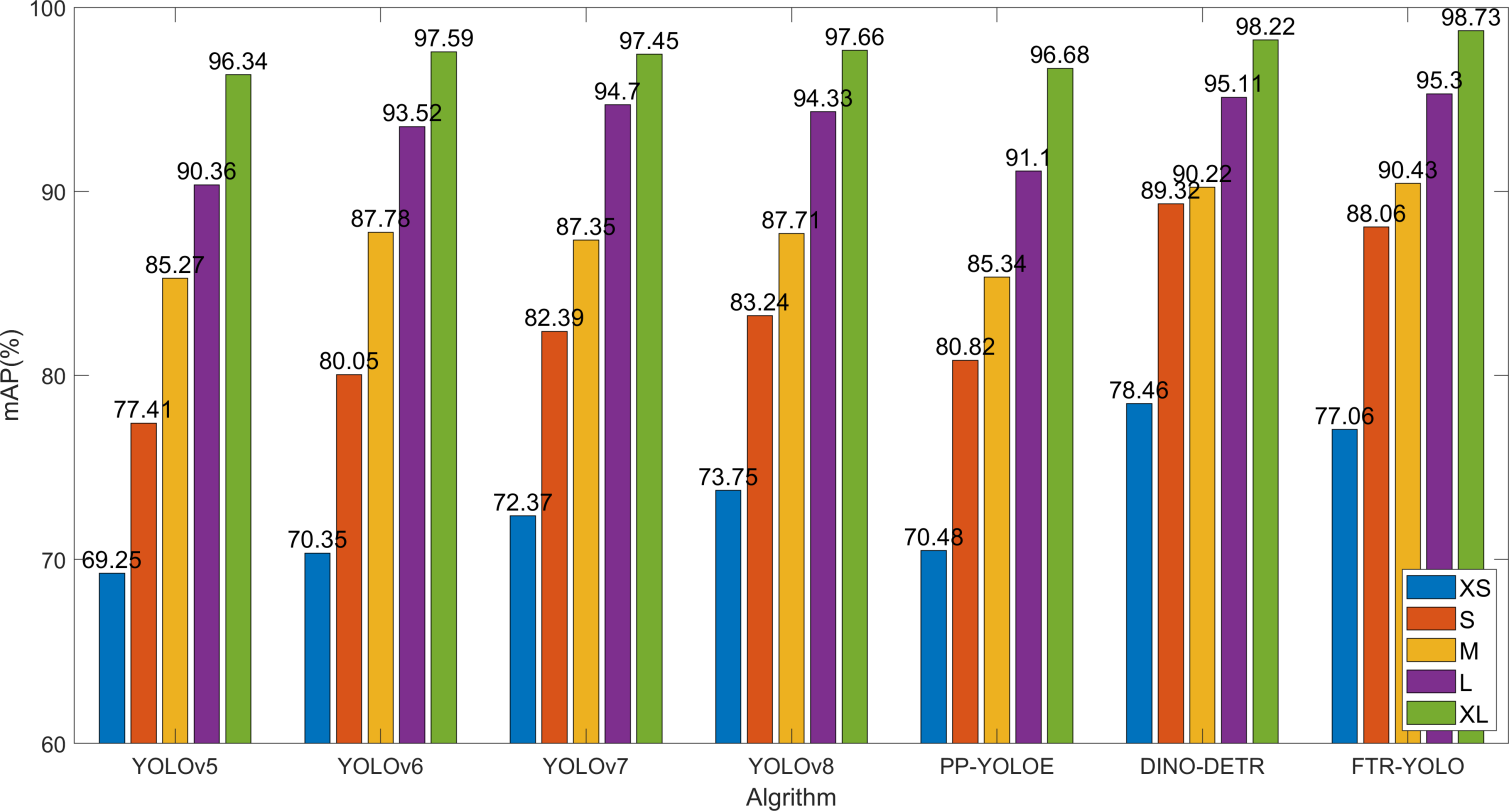
Object size sensitivity analysis.

As shown in **Figure 7**, The YOLOv5 and PP-YOLOE perform well in large target region (XL and L), but the detection effect of small target decreases sharply (XS and S). YOLOv6, v7, and v8 have shown slight improvements in detection accuracy compared to YOLOv5. Among them, v8 performs better on smaller scales (XS and S) while demonstrating similar detection effectiveness on M, L, and XL scales. The DINO-DETR is optimal in the detection accuracy on smaller scales (XS and S). FTR-YOLO demonstrates superior performance on M (90.43%), L (95.30%), and XL (98.73%) scales. The mAP values show a significant improvement when compared to the other five YOLO algorithms on both XS and S scales. Specifically, it shows improvements of 7.81%/6.71%/4.69%/3.31%/6.58% on XS scale, and improvements of 10.65%/8.01%/5.67%/4.82%/7.24% on S scale. These improvements highlight the effectiveness of the system in achieving higher mAP values compared to its counterparts. While it may have slightly lower performance than DINO-DETR, FTR-YOLO is still the optimal choice due to its lightweight and real-time capabilities.

### 3.6 Image size sensitivity analysis

The Batch Random Resize is applied to a batch of images, which helps increase the diversity and randomness of the data. By introducing such variations during training, the model becomes more robust and better able to generalize to unseen examples. This technique can contribute to improving the overall performance and generalization ability of the model in tasks such as object detection or image classification. In our experiment, the data were randomly resized into the following 10 different sizes: [320, 384, 448, 480, 512, 544, 576, 640, 672, 704, 736, and 768].

**Figure 8:**
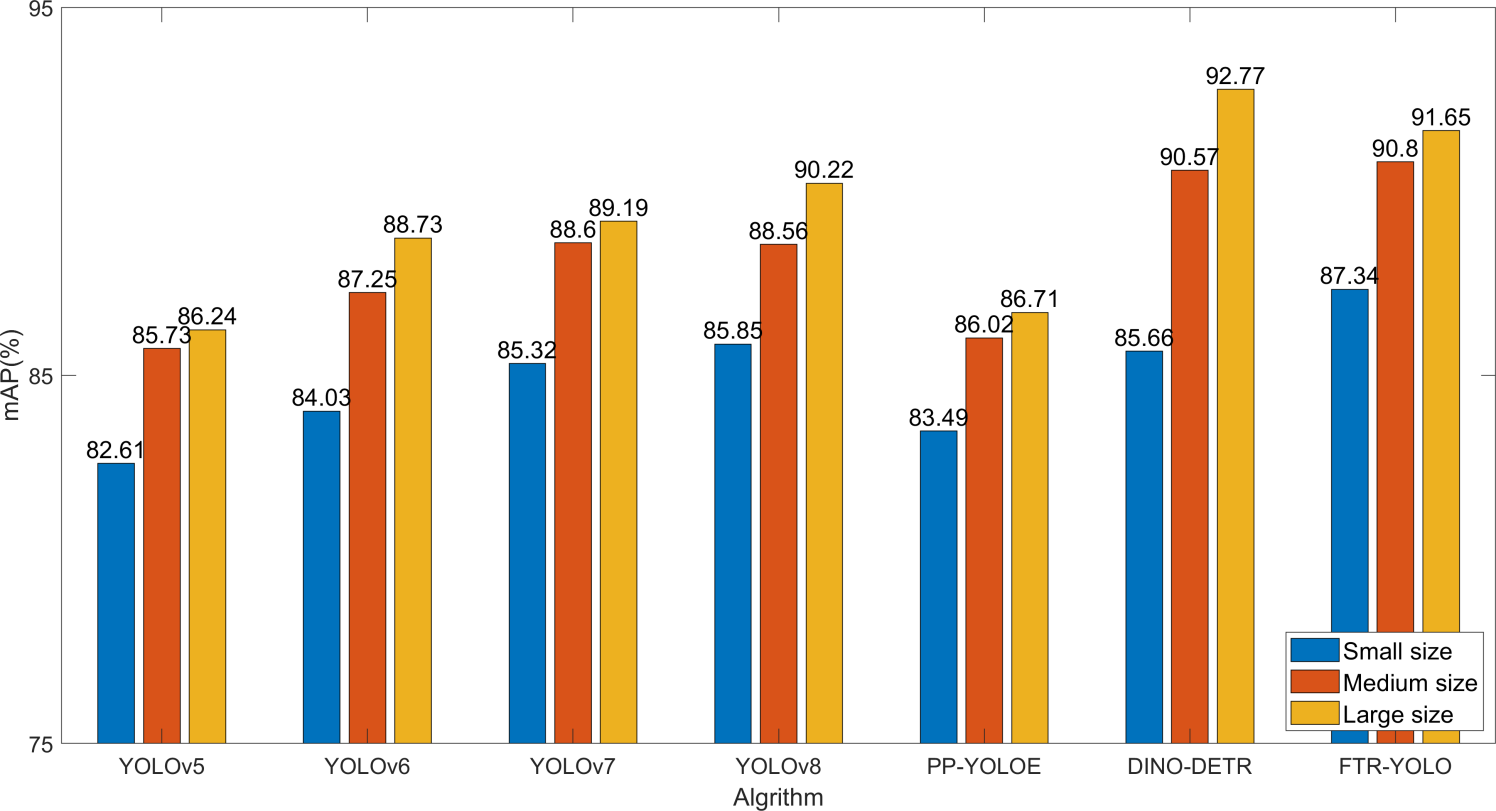
Image size sensitivity analysis.

To further validate the detection performance on images of varying sizes, we categorized the dataset into three groups based on different sizes: (1) small size, less than or equal to 480; (2) medium size, ranging from 480 to 768; (3) large size, greater than 768. **Figure 8** shows the detection performance of seven different algorithms.

The detection accuracy among samples of different sizes does not show significant variation, as illustrated in **Figure 8**. However, it should be noted that the detection accuracy is affected by the distortion introduced when resizing small-sized images to 640. Among the various algorithms, the DINO-DETR algorithm is particularly sensitive to this impact. On the other hand, FTR-YOLO demonstrates superior performance on small-sized images (87.34%) and medium-sized images (90.80%). Additionally, FTR-YOLO significantly improves mAP values compared to the other five YOLO algorithms on small-sized images by 4.73%, 3.31%, 2.02%, 1.49%, and 3.85%. It also improves mAP values on medium-sized images by 5.07%, 3.55%, 2.20%, 2.24%, and 4.78%. Furthermore, it improves mAP values on large-sized images by 5.41%, 2.92%, 1.46%, 1.34%, and 4.85%. Although FTR-YOLO may have slightly lower performance than DINO-DETR on large-sized images, it is still considered the optimal choice due to its lightweight design and real-time capabilities.

Based on the comparative evaluation in **Sections 3.4–3.6**, LH-VoVnet-39 outperforms YOLO’s backbone CSPDarknet-53 or CSPResnet-50, which replaced the convolution downsampling operation with LDS, enabling the model to better preserve important features. Additionally, the GC-RE-OSA module, along with residual connections and eSE attention mechanism, further improves feature extraction. Furthermore, we have made improvements to the TAP and loss selection based on YOLOv7 and v8 decoupled heads. As a result, FTR-YOLO demonstrates superior performance in terms of mAP and AP values for each category, with minimal numerical differences and strong generalization capabilities (**Table 8**).

Due to VoVnet-39 having fewer layers and the utilization of lightweight ghost modules instead of convolutions, in addition to a real-time transformer that consists of 2D position embedding and a single-scale Transformer encoder, but does not include decoder, FTR-YOLO achieves comparable FPS performance to YOLOv8 while delivering optimal results (**Table 8**).

On the other hand, DINO-DETR, with its multi-scale Transformer encoder and decoder, possesses more input feature maps and layers, resulting in better performance for object detection. It outperforms FTR-YOLO in specific metrics such as mAP in **Table 8**, mAP for XS and S object scales in **Figure 7**, and mAP for large-sized inputs in **Figure 8**. However, this improvement comes at the cost of significantly increased computational complexity, leading to an FPS of only 2, which limits its practical applications.

### 3.7 Performance visualization on FTR-YOLO

**Figure 9:**
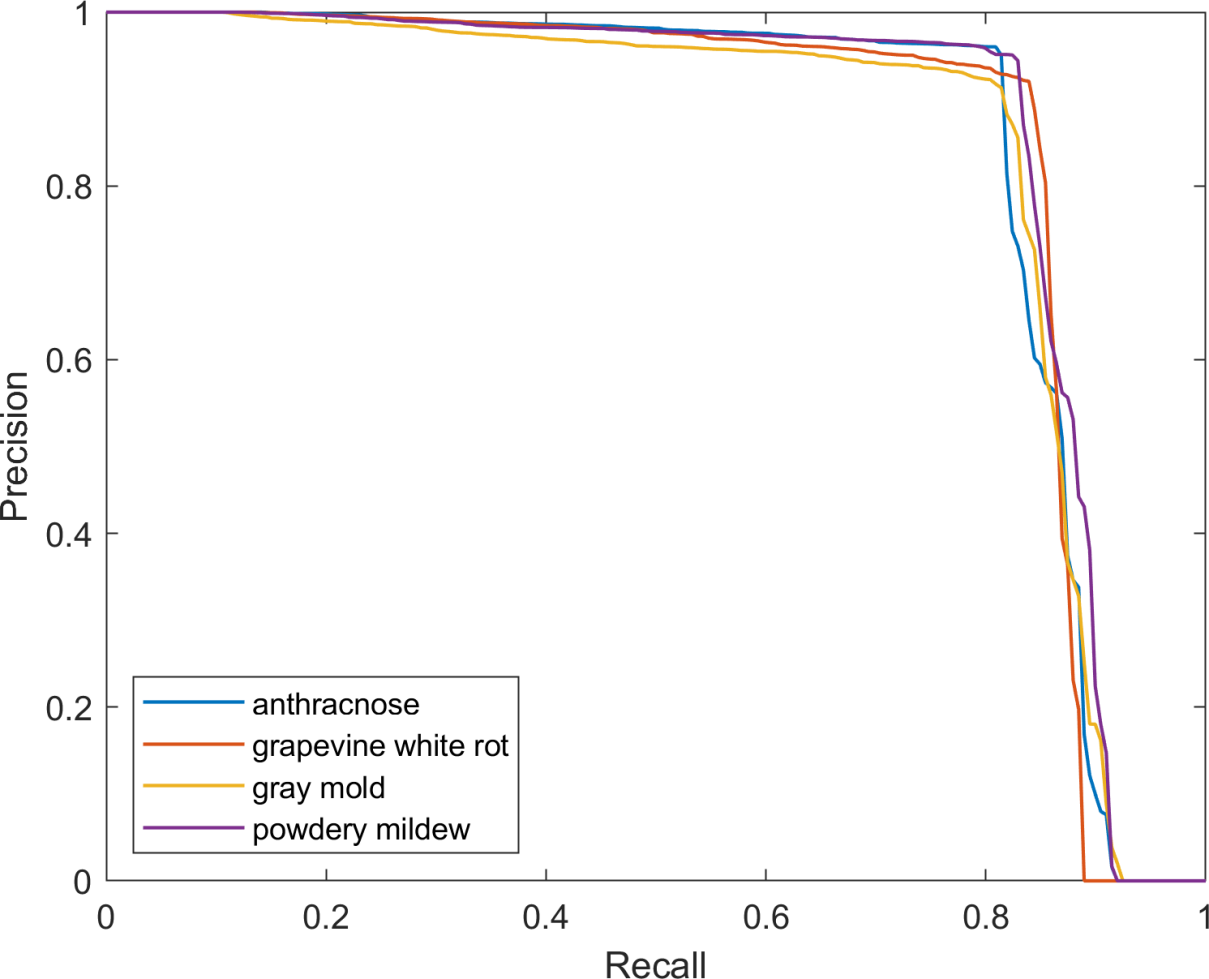
The p–r curve of FTR-YOLO.

The precision–recall curves of each disease are provided in **Figure 9**, which intuitively shows the detailed relationship between precision and recall. It has been observed that as recall increases, the rate of change in precision also increases. When the graph’s curve is closer to the upper right corner, it indicates that the drop in precision as recall increases is less noticeable, indicating improved overall performance of FTR-YOLO.

**Figure 10:**
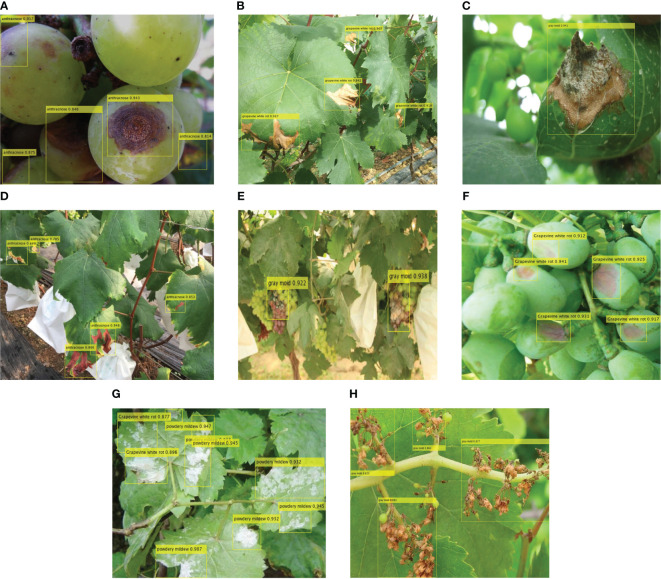
The detection results of FTR-YOLO. **(A)** diseased leaves of anthracnose, **(B)** diseased leaves of grapevine white rot, **(C)** diseased leaves of gray mold, **(D)** diseased leaves of powdery mildew, **(E)** diseased fruits of gray mold, **(F)** diseased fruits of grapevine white rot, **(G)** diseased fruits of anthracnose, and **(H)** diseased inflorescence of gray mold.

**Figure 11:**
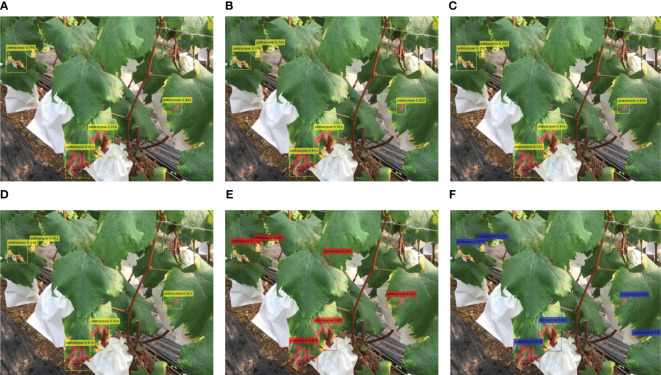
The detection results of different methods. **(A)** YOLOv5, **(B)** YOLOv6, **(C)** YOLOv7, **(D)** YOLOv8, **(E)** PPYOLO-E, and **(F)** DINO-DETR.

The detection results of four diseases of grape are shown in **Figure 10**. **Figures 10A–D** show the detection results of diseased leaves of anthracnose, grapevine white rot, gray mold, powdery mildew, respectively, while **Figures 10E–G** show the detection results of diseased fruits of gray mold, grapevine white rot and anthracnose respectively. **Figure 10H** shows the diseased inflorescence of gray mold. The results indicate that the FTR-YOLO model exhibits precise detection of diverse symptoms in different parts of the vine within natural scenes. This underscores the model’s remarkable generalization and robustness. It is evident that the majority of detection boxes have scores exceeding 0.8. Additionally, a substantial portion of the diseased areas have been accurately detected, highlighting the exceptional precision and precise localization capabilities of the proposed model. We also compared the detection performance of different algorithms. For details, please see **Figure 11**.

The experimental results in **Figure 11** show that YOLOv5 missed some small objects, while the PPYOLOE and DINO-DETR algorithms detected additional object areas. There are slight differences in the detected bounding boxes and confidence levels among the different algorithms, which overall align with the experimental results obtained in the paper. The proposed FTR-YOLO (**Figure 10A**) performs well in terms of detection accuracy and confidence levels.

## 4 Discussions

Based on the information provided, the FTR-YOLO model is proposed in this paper to achieve accurate, real-time, and lightweight intelligent detection of four common grape diseases in natural environments. The model incorporates several improvements in its components. In backbone, the LH-VoVNet is introduced, which includes LDS layer and Ghost-conv. Additionally, eSE blocks and residual connections are added to the OSA module (GC-RE-OSA module). Experimental results presented in **Table 5** demonstrate that the LH-VoVNet achieves optimal performance in terms of detection (mAP 86.79%), lightweight design (Params 24.7MB), and real-time capabilities (FPS 56). The neck component also undergoes improvements. Only the C5 feature map output by the backbone is selected as the input for the real-time Transformer, includes 2D position embedding and SSTE. Additionally, the C3 module is replaced with the GC-RE-OSA module in PAN + FPN. Experimental results presented in **Table 6** show that the improved neck further enhances performance in detection (mAP 88.85%) and lightweight design (Params 22.5MB). In the head and loss component, the ITAP is proposed, and TAL is used with VFL and DFL. Experimental results presented in **Table 7** demonstrate that the ITAP Decoupled Head + TAL achieves an optimized mAP of 90.67%. Moreover, **Table 8**; **Figures 7**, **8** show the superior performance of FTR-YOLO compared to state-of-the-art YOLO detectors in terms of accuracy (mAP 90.67%), speed (FPS 44), and lightweight design (Params 24.5MB), particularly improved accuracy on smaller scales (XS and S) and different sample sizes.

## 5 Conclusion and future works

In this paper, we propose a real-time and lightweight detection model, called Fusion Transformer YOLO, for grape disease detection. In backbone, we integrate GC-RE-OSA module based on VoVnet, effectively improving the ability of network to extract feature information while keeping the network lightweight. In neck component, an improved Real-Time Transformer with 2D position embedding and SSTE are incorporated to the last feature map to accurate detection of small targets in natural environments. In head component, the Decoupled Head based on the ITAP is adopted to optimize detection strategy. Our proposed FTR-YOLO achieved 24.5MB Params, 90.67% mAP@0.5 with 44 FPS, which outperformed YOLOv5-v8 and PP-YOLOE. Although FTR-YOLO uses a real-time Transformer to improve model performance, it still falls behind DETR in terms of performance due to DETR’s multi-scale and multi-layer global transformer architecture.

Future studies plan to explore the fusion of CNN and transformer models, as well as the integration of multimodal features, to further enhance the model’s performance. Additionally, this paper focuses on disease detection in grapes, theoretically, the FTR-YOLO algorithm has the potential to achieve good performance when retrained on other datasets. It can be applied to tasks such as the detection of plant traits and pest diseases in other plants.

## Data availability statement

The original contributions presented in the study are included in the article/supplementary material. Further inquiries can be directed to the corresponding author.

## Author contributions

YL: Conceptualization, Methodology, Software, Writing – original draft, Writing – review & editing. QY: Data curation, Funding acquisition, Writing – review & editing. SG: Supervision, Validation, Visualization, Writing – review & editing.
